# Atrial Leadless Pacemaker Embolization to the Left Common Iliac Vein With Successful Retrieval: A Case Report

**DOI:** 10.1002/joa3.70393

**Published:** 2026-06-10

**Authors:** Toshihiko Goto, Masashi Yokoi, Yoshiro Tsuruta, Kento Mori, Yoshihiro Seo

**Affiliations:** ^1^ Department of Cardiology Nagoya City University Graduate School of Medical Sciences Nagoya Japan

**Keywords:** atrial leadless pacemaker, dislodgement, extracardiac embolization, migration, pacemaker retrieval

## Abstract

A rare extracardiac complication in which a dislodged atrial leadless pacemaker migrated retrogradely to the left common iliac vein. This case highlights the role of venous anatomy in implantation difficulty and demonstrates successful device retrieval using a preventive strategy.

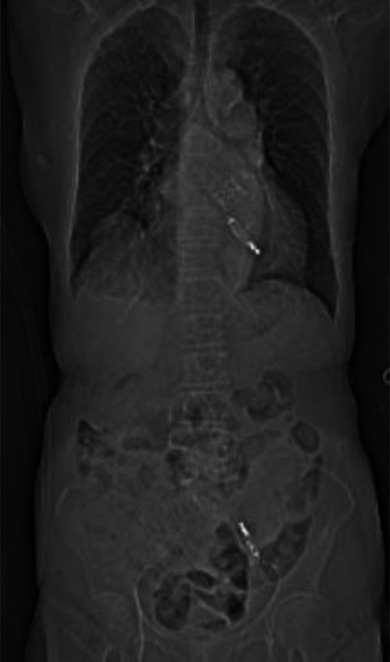

An 86‐year‐old woman with a history of percutaneous coronary intervention, transcatheter aortic valve implantation, and left atrial appendage closure had undergone transvenous dual‐chamber pacemaker implantation via the right subclavian vein 1 year earlier for paroxysmal atrioventricular block, with the right‐sided approach chosen because of prior left breast cancer surgery with lymph node dissection more than 10 years earlier. The system was explanted early because of infection, and she subsequently underwent implantation of a leadless ventricular pacemaker (Aveir VR, Abbott, Chicago, IL) for backup pacing, given the low ventricular pacing burden (3%) at the time of extraction. Although three screwing attempts were performed, the initial pacing threshold was high (1.5 V at 1.0 ms). Her pacing burden, initially low, progressively increased to nearly 100%, resulting in accelerated battery depletion with an estimated remaining battery life of approximately 1 year at the one‐year follow‐up. Continuous ventricular pacing in VVI mode also contributed to worsening heart failure symptoms, and she was referred for device replacement and implantation of an atrial leadless pacemaker. She continued to prefer a leadless system rather than conduction system pacing via a left‐sided transvenous approach related to prior breast cancer surgery. At the time of the second implantation, left ventricular ejection fraction was preserved (63.7%); however, left ventricular dyssynchrony was observed. Plasma B‐type natriuretic peptide increased from 388.2 to 1148.6 pg/mL. Despite moderate tricuspid regurgitation, the pressure gradient was elevated (41.5 mmHg; velocity 3.2 m/s) on echocardiography, and right atrial morphology is shown on computed tomography (Figure S1).

For the second leadless implantation, the initial leadless pacemaker was extracted due to concerns about procedural interference and patient preference. Because of right femoral vein stenosis (Figure 1A; Figure S2A,B), the procedure was performed via the left femoral vein. The ventricular leadless pacemaker was successfully extracted, and a new ventricular leadless pacemaker (Aveir VR) was implanted. Post‐implant measurements showed no R‐wave, with a pacing threshold of 0.75 V at 0.4 ms and an impedance of 510 Ω after two screwing attempts.

**FIGURE 1:**
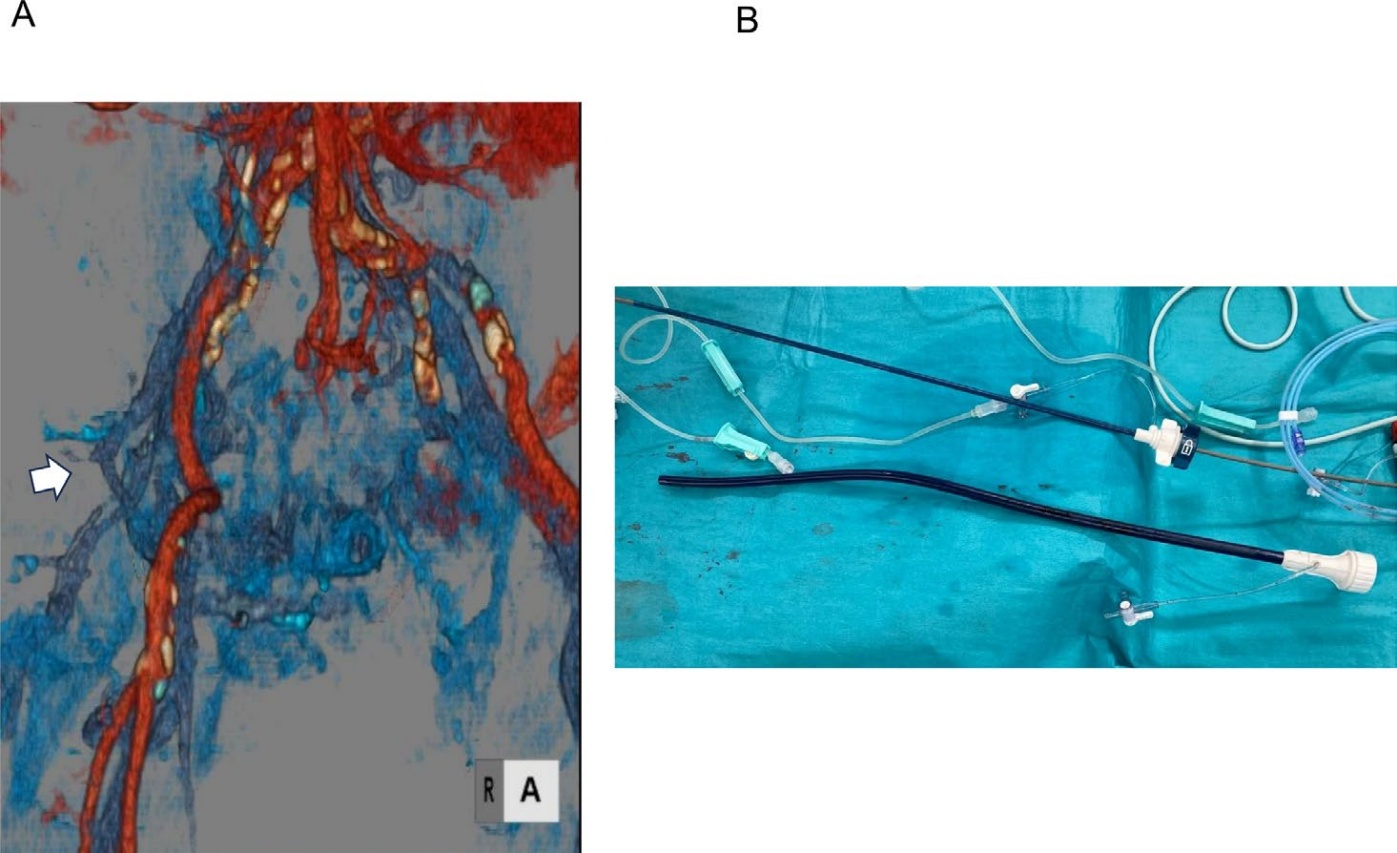
(A) Post‐procedural computed tomography (CT) obtained after two large‐bore sheath insertions demonstrated significant stenosis of the right common iliac vein (white arrow), corresponding to the venous narrowing that had been encountered intraoperatively. (B) The delivery sheath is shown after completion of the atrial leadless pacemaker implantation via the left femoral vein. Marked bending and curvature of the sheath are visible, reflecting the technical difficulty of the procedure.

We then implanted an atrial leadless pacemaker (Aveir AR) via the left femoral vein, which proved technically challenging. The delivery sheath after implantation demonstrated marked bending and curvature (Figure 1B), reflecting procedural difficulty. The right atrium was suspected to be globally diseased, making it difficult to obtain a stable and adequate wave amplitude, with intracardiac electrogram amplitudes < 1.0 mV at multiple sites. Although the current of injury was not robust, intracardiac electrograms showed a slight increase in S‐wave amplitude toward baseline after screwing compared with the pre‐screw S‐wave amplitude (Figure 2A,B), accompanied by an increase in impedance (pre‐screw 220 Ω; tether mode 240 Ω). Unlike ventricular leadless pacemakers, where impedance elevation indicates adequate fixation, impedance changes in atrial implantation are less pronounced and should be interpreted with other parameters [1]. The pacing threshold decreased 10 min after tethering (2.75 V/0.4 ms, 1.5 V/1.5 ms) compared with immediately post‐tethering (4.0 V/0.4 ms, 2.75 V/1.5 ms). The difference in thresholds between the two pulse widths (0.4 ms and 1.5 ms) at 10 min was 1.25 V (2.75–1.5 V). As no more favorable fixation site was achievable, these electrical findings were considered acceptable, consistent with previous reports [2] showing that elevated acute thresholds may decrease after implantation, and the Aveir AR was implanted near the base of the right atrial appendage after four screwing attempts (Figure 2C,D; Figure S3). Fixation was confirmed by a tension test. The system was programmed for DDD pacing, and bidirectional wireless communication between the Aveir AR and Aveir VR was satisfactory.

**FIGURE 2:**
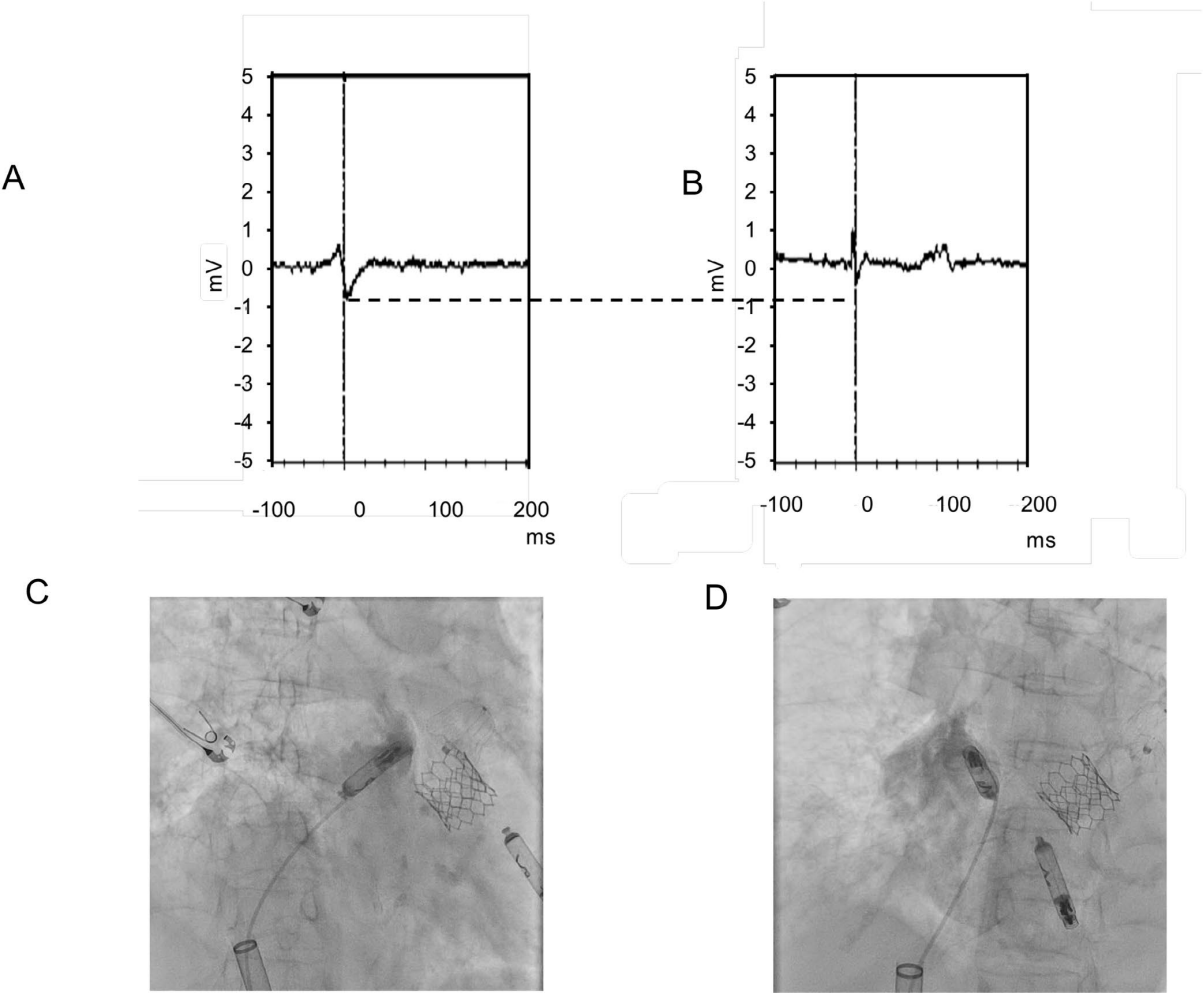
(A) Intracardiac electrogram before screwing (pre‐screw) and (B) after screwing. Compared with the pre‐screw tracing, the S‐wave amplitude after screwing became slightly closer to the baseline. A dotted line connecting the pre‐screw and post‐screw S waves is shown for comparison. (C, D) Right atrial angiography obtained after atrial leadless pacemaker implantation. (C) Right anterior oblique 30° view and (D) left anterior oblique 30° view demonstrate the device positioned near the base of the right atrial appendage.

However, DDD pacing was not observed on continuous monitoring from the night of the procedure, and the atrial leadless pacemaker was not visible on chest radiography the following day. Computed tomography revealed embolization of the atrial leadless pacemaker into the left common iliac vein (Figure 3A–C). An urgent retrieval procedure was performed. A Berman angiographic balloon catheter (Teleflex Inc., Wayne, PA, USA) was advanced via the right internal jugular vein and inflated centrally to prevent proximal migration (Figure 4A). An Aveir introducer was inserted via the left femoral vein, and the distally positioned docking button was grasped with an Amplatz GooseNeck snare (Figure 4B) and withdrawn into the sheath, which was then carefully removed from the body. Completion angiography confirmed the absence of vascular injury. Given the technical difficulty of the initial implantation, repeat atrial leadless pacemaker implantation was deferred.

**FIGURE 3:**
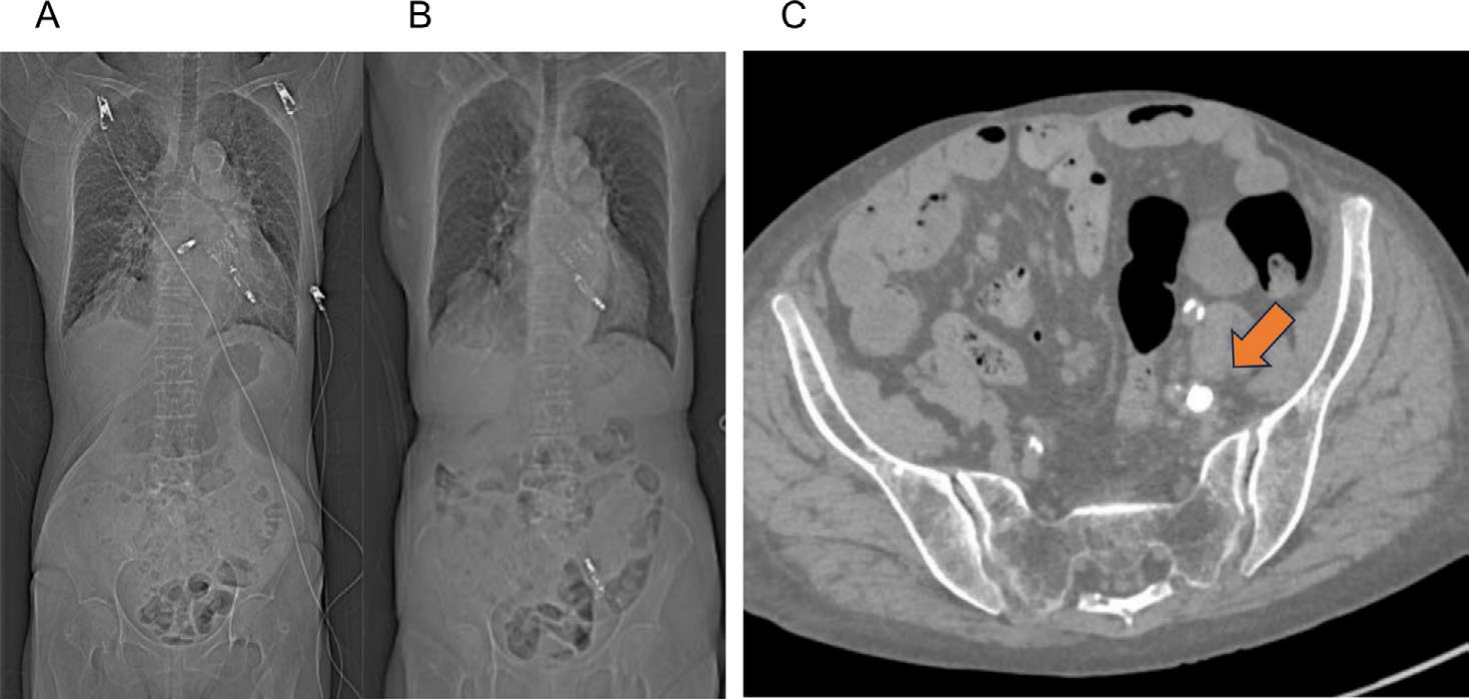
Imaging findings of atrial leadless pacemaker embolization. (A) CT scan confirming the atrial leadless pacemaker in the right atrium. (B) CT showing migration of the device into the left lower extremity. (C) Axial non‐contrast CT demonstrating the atrial leadless pacemaker lodged in the left common iliac vein (orange arrow).

**FIGURE 4:**
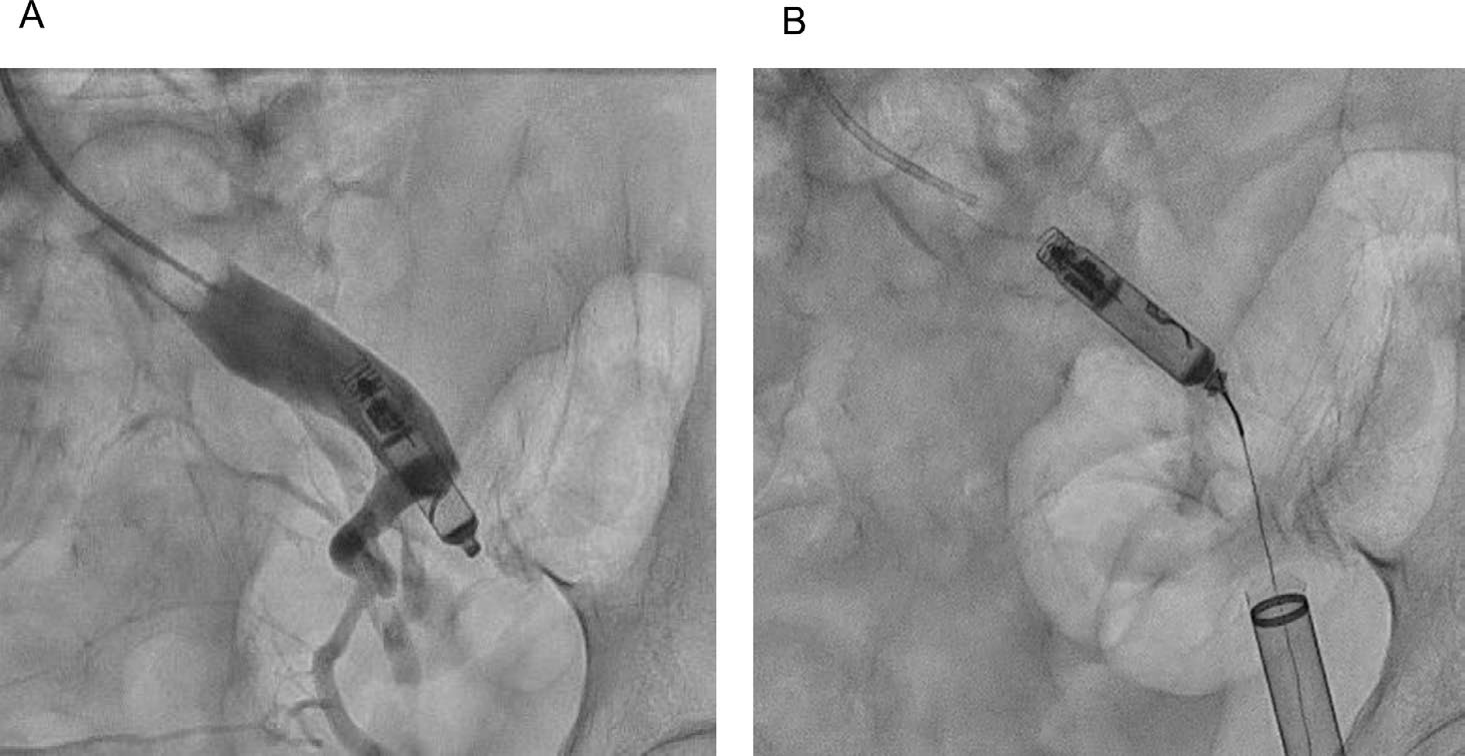
(A) A Berman angiographic balloon catheter was advanced via the right internal jugular vein and inflated centrally to prevent proximal migration of the atrial leadless pacemaker. (B) The dislodged atrial leadless pacemaker was successfully engaged using an Amplatz Goose Neck snare.

This case represents a rare instance of extracardiac embolization of an atrial leadless pacemaker to the left common iliac vein. Dislodgement of leadless pacemakers is uncommon [3] and usually occurs acutely because of inadequate initial fixation [4]; to date, reported embolization sites have been limited to the right ventricle or pulmonary artery [5]. In this case, loss of atrial capture on the night of implantation indicates acute dislodgement related to suboptimal fixation during a technically challenging left femoral approach.

Although the precise mechanism of extracardiac embolization of an atrial leadless pacemaker remains unclear, the device may have stayed near the inferior vena cava ostium while the patient was supine and later migrated under gravitational forces triggered by postural changes or intrathoracic pressure increases (e.g., coughing or straining). Relatively elevated right‐sided pressures may also have contributed to extracardiac embolization.

This case also highlights a preventive retrieval strategy. Proximal balloon occlusion using a Berman catheter likely enhanced procedural safety and ensured successful retrieval. Thrombus formation at the embolization site further underscores the need for careful postprocedural surveillance and short‐term anticoagulation.

Right femoral vein stenosis necessitated a left femoral approach, possibly contributing to suboptimal fixation of the atrial leadless pacemaker. The stenosis likely resulted from prior large‐bore sheath use (14‐Fr for left atrial appendage closure and 27‐Fr for leadless pacemaker implantation within 3 months), highlighting potential challenges with repeated large‐bore access.

## 1 Conclusion

We report a rare case of atrial leadless pacemaker embolization into the left common iliac vein, a previously unrecognized complication that was successfully managed with a preventive retrieval strategy.

## Funding

The authors have nothing to report.

## Ethics Statement

This case report complies with the ethics and integrity policies of the *Journal of Arrhythmia*.

## Consent

Written informed consent was obtained from the patient for publication of this case report and accompanying images.

## Conflicts of Interest

The authors declare no conflicts of interest.

## Supporting information


**Figure S1:** Post‐implant chest computed tomography (axial view) showing right atrial enlargement. The orange line indicates the tricuspid annular diameter, which measured 41 mm.

**Figure S2:** Three‐dimensional computed tomography reconstruction of the venous anatomy. (A) Left‐sided view and (B) right‐sided view. The veins are shown in blue, and the arteries are shown in red. The left‐sided venous system demonstrates marked tortuosity compared with the relatively straight course on the right side. Orange arrows indicate the direction and curvature of the venous pathway.

**Figure S3:** Postoperative computed tomography showing the atrial leadless pacemaker positioned at the base of the right atrial appendage (orange arrow).

## Data Availability

Data sharing is not applicable to this article as no datasets were generated or analyzed.
