# Supplementary figures and images for: Atrial Leadless Pacemaker Embolization to the Left Common Iliac Vein With Successful Retrieval: A Case Report
Source: J Arrhythm. 2026 Jun 10;42(3):e70393. doi: 10.1002/joa3.70393 (PMC13250470; doi:10.1002/joa3.70393)

## Slide 1
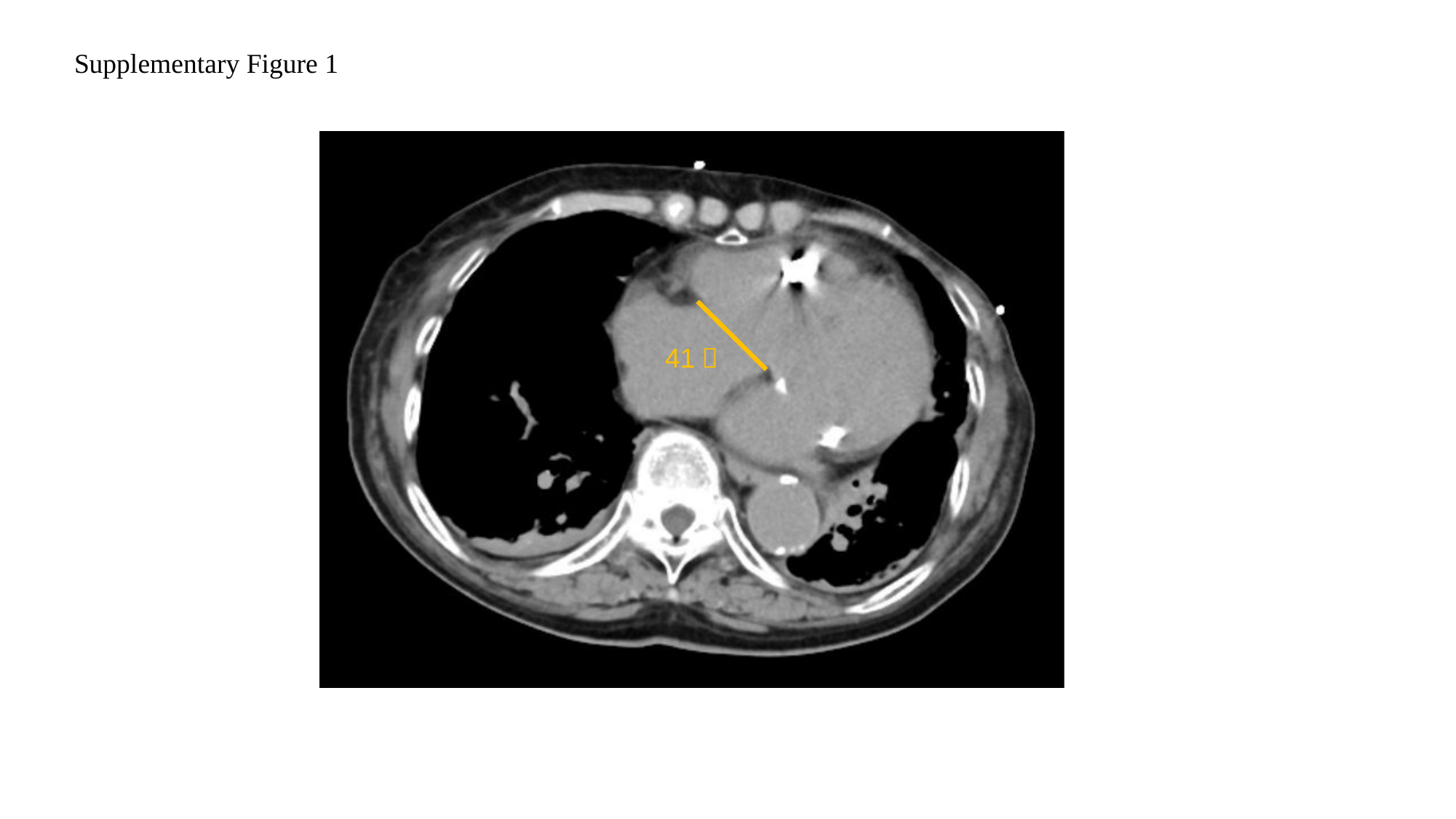

Supplementary Figure 1
41㎜

Supplement: Supplementary file 1 — Figure S1: Post‐implant chest computed tomography (axial view) showing right atrial enlargement. The orange line indicates the tricuspid annular diameter, which measured 41 mm. [file JOA3-42-e70393-s001.pptx]

## Slide 1
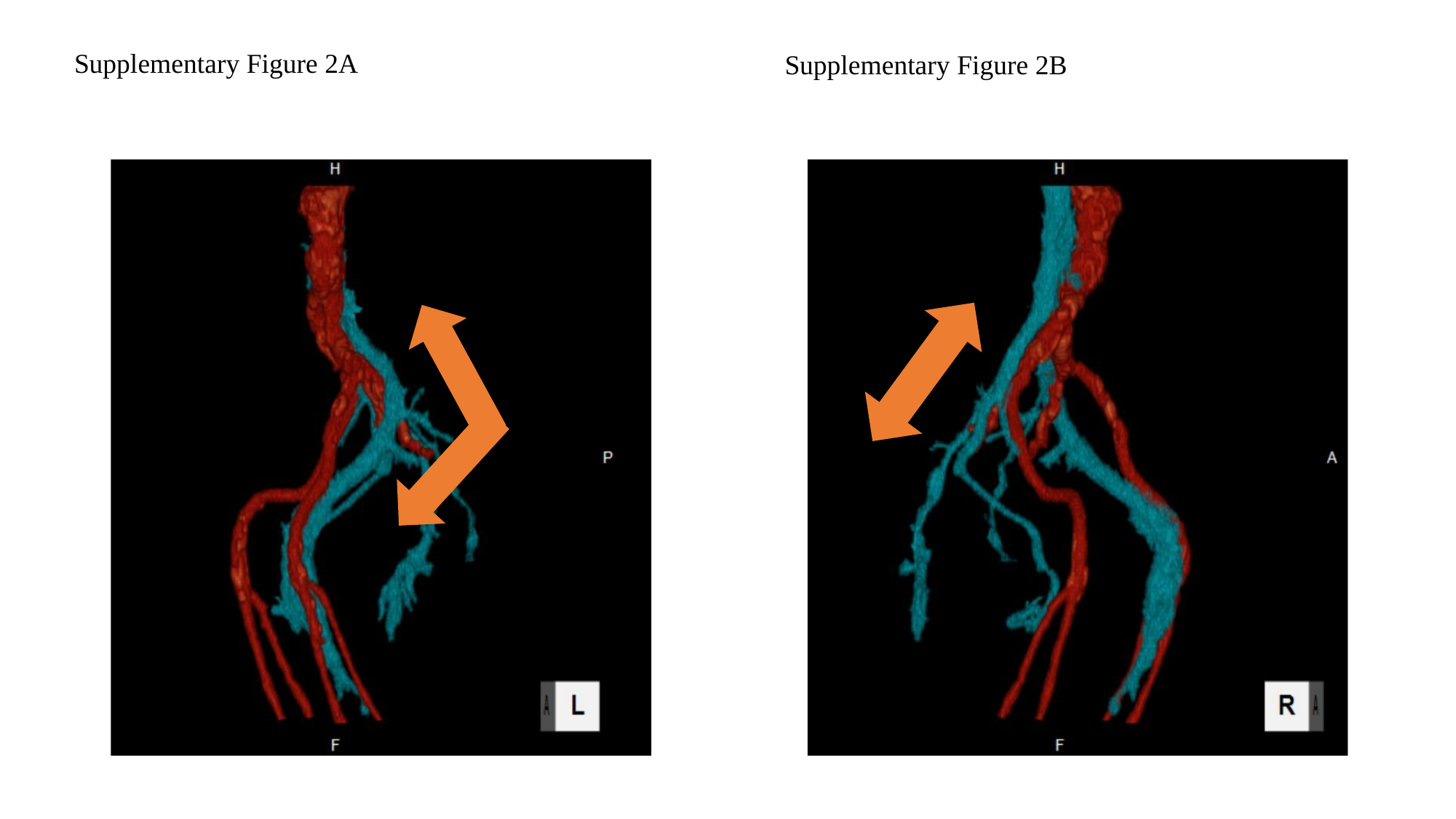

Supplementary Figure 2A
Supplementary Figure 2B

Supplement: Supplementary file 2 — Figure S2: Three‐dimensional computed tomography reconstruction of the venous anatomy. (A) Left‐sided view and (B) right‐sided view. The veins are shown in blue, and the arteries are shown in red. The left‐sided venous system demonstrates marked tortuosity compared with the relatively straight course on the right side. Orange arrows indicate the direction and curvature of the venous pathway. [file JOA3-42-e70393-s002.pptx]

## Slide 1
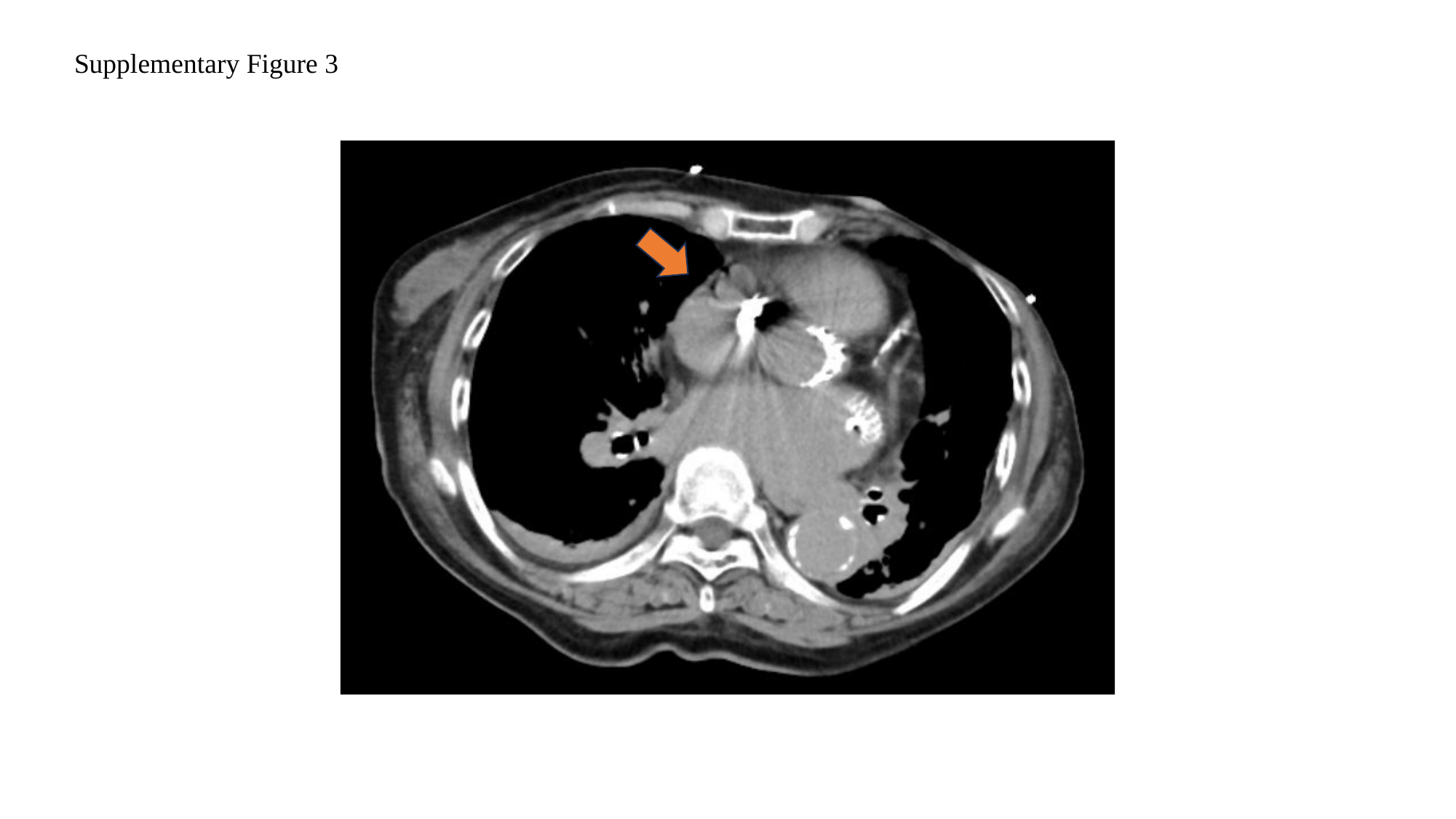

Supplementary Figure 3

Supplement: Supplementary file 3 — Figure S3: Postoperative computed tomography showing the atrial leadless pacemaker positioned at the base of the right atrial appendage (orange arrow). [file JOA3-42-e70393-s003.pptx]
